# Combined Hot Air, Microwave, and Infrared Drying of Hawthorn Fruit: Effects of Ultrasonic Pretreatment on Drying Time, Energy, Qualitative, and Bioactive Compounds’ Properties

**DOI:** 10.3390/foods10051006

**Published:** 2021-05-04

**Authors:** Yousef Abbaspour-Gilandeh, Mohammad Kaveh, Hamideh Fatemi, Muhammad Aziz

**Affiliations:** 1 Department of Biosystems Engineering, College of Agriculture and Natural Resources, University of Mohaghegh Ardabili, Ardabil 56199-11367, Iran; sirwankaweh@uma.ac.ir; 2 Department of Horticulture, College of Agriculture and Natural Resources, University of Mohaghegh Ardabili, Ardabil 56199-11367, Iran; ha.fatemi@yahoo.com; 3 Institute of Industrial Science, The University of Tokyo, 4-6-1 Komaba, Meguro-ku, Tokyo 153-8505, Japan

**Keywords:** *Crataegus aronia*, drying, specific energy, color, shrinkage, antioxidants

## Abstract

The present study aimed to examine the effect of ultrasonic pretreatment and hot air, microwave–hot-air, infrared–hot air, and freeze-drying on the drying time, specific energy (SE), qualitative properties (i.e., color, shrinkage, and rehydration ratio), and bioactive compounds’ properties (i.e., antioxidant activity, phenolic, and flavonoid contents) of hawthorn fruit. Drying of hawthorn was conducted from 45 min for the ultrasonic + microwave–hot-air drying to 1280 min for the freeze-drying method. The lowest amount of SE was obtained using the ultrasonic-microwave–hot-air drying method, which was 47.57 MJ/kg. The lowest values in color changes (12.25) and shrinkage (17.21%) were recorded for the freeze-drying method, while the highest amounts for these traits were 45.57% and 66.75% in the HA drying, respectively. In general, the use of different drying methods reduces the antioxidant capacity (AC), total phenolic content (TPC), and total flavonoid content (TFC) during processing compared to fresh samples. The highest values for AC, TPC, TFC, and the rehydration ratio were 30.69%, 73.07 mg-GAE/gdw, 65.93 mg-QE/gdw, and 2.02 for the freeze-drying method, respectively.

## 1. Introduction

Hawthorn, scientifically known as *Crataegus aronia*, is a deciduous tree or shrub belonging to the Rosaceae family. To date, approximately 21 species of this type have been identified in Iran. This plant has high medicinal and nutritional value and has long been used to treat various human diseases including liver diseases, hypertension, heart diseases, and stress [1]. The chemical compounds of hawthorn include flavonoids, triterpene acids, proanthocyanins, and organic acids [2,3].

Owing to the growing global population and declining sources of non-renewable energy, the effort to use new non-destructive technologies to improve the quality of human life has become a matter of great concern to scientists and researchers. Saving energy consumption, improving the quality of products, increasing the rate, and reducing the processing time are the parameters often considered for the application of new technologies [4].

The high moisture content of fruits plays an important role in their storage. The moisture reduction or drying through the simultaneous transfer of mass and heat is widely used to increase the shelf life, facilitate transportation, maintain quality, and reduce the post-harvest loss of agricultural products to produce dried fruits and vegetables [5]. Drying of foods, especially fruits, can be performed in various ways, including solar drying, hot air, microwave, infrared, freeze, and those combined drying methods such as microwave–hot-air and infrared–hot air. Hot air drying is one of the oldest and most common thermal methods. The mass and heat transfer occurs along with a phase shift; therefore, it is a very expensive method from the perspective of energy consumption [6,7]. Freeze drying is one of the best drying methods to maintain the quality of agricultural products. The main advantages of the freeze-drying method are the high quality of the product and the preservation of its structure [4,8]. In addition to the advantages of using the freeze-drying method, the high energy consumption needed for freezing the product and creating the vacuum are some of the disadvantages of using this method [9,10].

Nowadays, other methods combining different types of drying methods to increase the efficiency have been developed. Pretreatment is one of the new methods used to dry agricultural products. Pretreatment can reduce texture water and facilitate mass transfer during air drying [11]. Various pretreatment methods, such as ultrasonic wave, blanching, microwave, and citric acid, are used for food drying. The use of ultrasonic wave as a new pretreatment for food drying has received interest in recent years [12]. Ultrasonic wave began to be used in the years before World War II with the applications in processes, such as surface cleaning and extraction, and this has continued to the present day. In recent decades, the use of ultrasonic waves in the food industry has grown significantly. The applications of the ultrasonic waves in this field include quality assessment, process monitoring, defoaming, degassing, extraction, and drying [13]. The use of ultrasonic waves as a complement to the classical methods of drying with hot air, microwave, and infrared and combined methods increases the moisture diffusivity and reduces the drying time. This method reduces the costs of product manufacturing due to the reduction of drying time [14]. Applying the pretreatment before drying using ultrasonic wave involves immersing the food in water or in a hypertonic aqueous phase [15]. The results of the research on the drying process of some fruits and vegetables using the waves have shown that they have a significant effect on the increased rate of mass and heat transfer due to the changes in the boundary layers, density, and diffusivity of materials. On the other hand, ultrasonic waves are able to remove moisture from materials without a significant increase in temperature [11,13]. Also, in this method, food is less damaged than other drying methods.

Many researchers have studied various drying properties, such as qualitative (color, shrinkage, rehydration ratio (RR), thermal (kinetics and specific energy) and nutritional (antioxidant capacity (AC), total phenolic content (TPC), and total flavonoid content (TFC)) properties and used ultrasonic pretreatment before different methods to dry agricultural products. For instance, Ren et al. [10] investigated the effect of blanching and ultrasonic pretreatments on the qualitative properties (TPC, TFC, and quercetin) of onion dried by hot-air and freeze-drying methods. The ultrasonic pretreatment for 1, 3, and 5 min and the blanching pretreatment with hot water at 70 °C for 1, 3, and 5 min were applied to the onion samples. The results showed that ultrasonic pretreatment led to a better maintenance of qualitative and antioxidant properties of dried onions in the hot-air and freeze methods. Also, comparing two methods of freeze and hot-air drying showed that the use of freeze-drying further maintained the AC and qualitative properties of the dried onions. In another study, Jiang et al. [16] evaluated the energy consumption, qualitative, and AC properties of okra during the drying process in a combined freeze–microwave–vacuum drying and compared them with other drying methods such as hot air flow, microwave–vacuum, freeze, and combined hot-air–microwave–vacuum. The results showed that the AC properties and color changes in the two methods of freeze and combined freeze–microwave–vacuum drying were significantly different from other methods. Also, the comparison of energy parameters and drying time between the two methods of freeze-drying and combined freeze–microwave–vacuum drying showed that the combined methods reduced both parameters. Motevali and Hashemi [17] examined the effect of freeze-drying using different pretreatments on the qualitative characteristics (i.e., RR, pH, vitamin C, failure force of samples, and overall color changes) of feijoa. The results showed that the highest and lowest pH values were obtained using potassium carbonate and ascorbic acid pretreatment and the highest and lowest rehydration occurred in microwave pretreatment and control treatment, respectively. The highest amount of vitamin C was obtained in the control sample, and the lowest amount was obtained in the treatment of blanching with hot water. Also, the highest and lowest color changes occurred using ascorbic acid and microwave pretreatment, respectively. In another study by Colucci et al. [18], the antioxidant properties of eggplant dried in a freeze-drying using ultrasonic pretreatment were investigated. The experiments were performed at several levels of temperature, air flow rate, and ultrasonic power. The results showed that increasing the temperature and air flow rate reduced the amount of antioxidant in the dried samples. The results also showed that in a constant sample size, the changes in ultrasonic pretreatment power had no significant effect on antioxidant properties. Dujmic et al. [19] studied the effect of high-intensity ultrasonic as a pretreatment on the drying time and texture characteristics of pear slices with different ultrasonic wave amplitudes. The results showed that the use of ultrasonic waves with different amplitudes was effective in shortening the drying time of pear slices. Increasing the ultrasonic wave intensity led to a decrease in the hardness of the dried samples. The sample elasticity was reduced at all ultrasonic wave amplitudes compared to the untreated samples. The study of Babagoltabar et al. [20] on the effect of using ultrasonic waves on the drying rate and the time required for tea leaves to reach the desired moisture in the warehouse conditions showed that the US wave had a significant effect on drying time, moisture content, and drying rate of fresh tea leaves at 5% probability level. Ultrasonic waves have a positive effect on the drying properties of cranberry snacks. According to the results, the use of ultrasonic waves reduced the drying time and color changes, while an increase was seen in the AC, TPC, and TFC [11].

The review of reported studies shows that the use of ultrasonic waves with different drying methods improves the performance indicators of the drying process in different products. Due to the medicinal importance of hawthorn fruit and the need for the proper processing, it seems that this technology can improve the quality of the final product while reducing the time and energy consumption in the drying process. Despite numerous reports on the successful use of ultrasonic waves for drying various products, no study has yet been reported on its effect on the thermal, qualitative, and bioactive compounds’ properties of hawthorn fruit. Therefore, in this study, hawthorn fruit dried by the hot-air, microwave–hot-air, infrared–hot-air, and freeze-drying methods using ultrasonic pretreatment before drying and the effects of each method on drying time, energy consumption, color, shrinkage, RR, AC, TPC, TFC, and pH of the dried samples were investigated.

## 2. Materials and Methods

### 2.1. Laboratory Materials

The harvesting of hawthorn fruit was performed in a manual and completely random manner from the trees in the gardens of Sardasht, located in West Azerbaijan Province, Iran, having a longitude of 45.53° east and a latitude of 36.25° north, in October 2020. All harvested samples were randomly selected, so that the fruits were healthy and free of any pests. To prevent fruit spoilage due to the high perishability of hawthorn fruit during the experiments, the fruits were stored in the refrigerator at 4 °C. The tested samples were transferred to the laboratory 2 h before the start of the experiment due to the temperature balance with the environment. The initial moisture content of the samples was calculated based on the ASABE standard by weighing method. For this purpose, the hawthorn fruit samples were dried at 70 °C for 24 h [1]. The initial moisture content of hawthorn fruit was 1.88 on dry basis (% dry basis.) [1]. The experiment was performed in three replications. Approximately 50 g of the sample was used in each experiment.

### 2.2. Ultrasonic Pretreatment

To apply ultrasonic pretreatment on the hawthorn fruit samples, an ultrasonic bath device (Parsonic 7500s, Parsnahand Co, Tehran, Iran) with a volume of 2.6 L, frequency of 37 kHz, and power of 70 W was used. Due to the constant frequency level of the device, the hawthorn fruit samples were placed in distilled water at 30 °C and were exposed to ultrasonic waves at a time level (20 min). To make the ultrasonic pretreatment operation uniform, the distilled water surface in the ultrasonic bath was filled to the recommended point.

### 2.3. Drying Types

#### 2.3.1. Hot-Air Drying

The dryer consists of a centrifugal fan to generate airflow (1 hp, 3000 rpm), an inverter (LS, Gyeonggi-Do, Korea) to control the airflow rate, three electrical elements to heat the air entering the dryer chamber with a weighing chamber, and several temperature sensors. An anemometer (Standard ST-8897, Kowloon, Hong Kong) was used to measure the airflow rate. The experiments were performed in this dryer at 60 °C and airflow rate of 1 m/s.

#### 2.3.2. Infrared-Hot-Air Drying

The dryer included a centrifugal fan (1 hp, 3000 rpm), airflow channel, tarpaulin connector, heating elements (three elements), directing tubes, infrared lamps (four pieces totally 1000 W), and infrared lamp housing. The infrared lamps were installed at a distance of 2 cm from the tray’s surface. The product tray consisted of a 25 cm circular perforated plate. Two K-type temperature sensors were used to measure the temperature. The drying experiments for drying hawthorn fruits were performed by the infrared–hot-air method at a power of 500 W and air temperature of 60 °C.

#### 2.3.3. Microwave–Hot-Air Drying

An axial blower (1 hp, 3000 rpm) was used to create air flow at different rates. Before entering the dryer chamber, air passed through three heating elements for heating, and two K-type temperature sensors (installed at the front-end of and inside the dryer chamber), thermostat (Atbin Mega, Tehran, Iran), and contactor were used to control the temperature of the hot air entering the dryer chamber. A magnetron (LG 2M246, Qingdao, China) with a maximum output power of 900 W was also used to generate microwaves. For more uniform distribution of the waves within the microwave chamber, one magnetron was used along the length of the instrument. The magnetrons’ feeding circuit included two transformers, one self-capacitor circuit, and one magnetron cooling system (two fans, P/N 2123XSL, China). The dryer chamber was a rectangle in the dimensions of a steel chamber with dimensions of 120 × 100 × 130 cm^3^. The hot air entered the dryer chamber from the lower part of the chamber. The hot-air stream passed through the product layer and, along with the water vapor evaporated from the drying of the product, exited through another duct installed at the top of the chamber. This device was designed to dry samples while the temperature and velocity of air and microwave power were controlled. The combined microwave–hot-air-drying experiments were performed at 450 W and 60 °C.

#### 2.3.4. Freeze Drying

A freeze dryer (FD-5003-BT, Dena Vacuum, Iran) was used to dry hawthorn fruits. The dryer has a condenser capacity of 3 L in 24 h at a temperature of −50 °C. To dry the samples, the dryer was set at air temperature −40 °C and vacuum pressure of 40 Pa. This device also included three trays for placing the product in bulk and vials and eight vacuum rubber valves for connecting volumetric flask, flask, Falcon, and Erlenmeyer flask. A digital scale (GF-600, Osaka, Japan) with a precision of ±0.001 g was used to measure the weight changes of the samples.

### 2.4. Specific Energy (SE)

The SE for drying hawthorn fruit in different drying methods was obtained using the method of Motevali and Tabatabaei [21] and Kaveh et al. [22] according to Equation (1):

(1) $$SE=\frac{E_{t}}{M_{w}}$$

where SE is the specific energy consumption (MJ/kg), $E_{t}$ is the energy consumption (kJ); $M_{w}$ is the final weight (kg).

The hawthorn samples were first pretreated by ultrasonic; then, they were dried by different techniques. Regarding the various power sources of the different applied methods, the equations used for the calculation of SE through ultrasonic pretreatment and the hot-air, infrared, microwave, and freeze dryers are listed in Table 1.

(2) $$EU_{ter}=A\cdot v\cdot \rho_{a}\cdot C_{a}\cdot \Delta t$$

(3) $$EU_{mec}=\Delta P\cdot M_{w}\cdot t$$

(4) $$EU_{ter}=A\cdot v\cdot \rho_{a}\cdot C_{a}\cdot \Delta t+K\cdot t$$

(5) $$EU_{ter}=(P\cdot t)\cdot 3600$$

(6) UP=U·I·cosϕ

(7) $$EU_{ter}=UP\cdot t$$

(8) $$EU_{freez}=E_{1}+E_{2}$$

where *EU_ter_* is the thermal energy consumption (kJ), *A* denote the tray area (m^2^), *v* represents the inlet air velocity (m/s), *C_a_* shows the specific heat (kJ/kg·°C), $\rho_{a}$ stands for air density (kg/m^3^), and Δ*T* and *t* are the temperature difference (°C) and drying time (h), respectively. Moreover, *EU_mec_* is the mechanical energy (kJ) and Δ*P* denotes the pressure difference (mbar). *M_w_* represents the weight loss (kg); *UP* is the ultrasound power (kW); *U* and *I* are also the applied voltage (V) and current (A) of the ultrasonic generator, respectively; *cos φ* is the power factor and equals to 0.8; *K* is the lamp power (W); and *E_1_* and *E_2_* are the vacuum pump and cooling system, respectively.

### 2.5. Quality Properties

#### 2.5.1. Color

The color of hawthorn fruit samples was measured using a colorimeter (HP-200, Shenzhen, China). The color test results included three Hunter indices (L, a, and b), where L denotes color whiteness (from L = 0 for black to L = 100 for white), a denotes green to red (from a = −60 for green to a = +60 for red), and b denotes blue to yellow (from b = −60 for blue to b = +60 for yellow) [24,25]. Before measuring the color of each sample, the device was calibrated using a standard white surface (L = 100). The color changes were calculated from Equation (9) [4].

(9) $$\Delta E=\sqrt{{\left(\Delta L^{*}\right)}^{2}+{\left(\Delta a^{*}\right)}^{2}+{\left(\Delta b^{*}\right)}^{2}}$$

where L* is the lightness, a* is the red - green, and b* is the yellow - blue.

#### 2.5.2. Shrinkage

Volume changes caused by the shrinkage of the product during the drying were obtained by the toluene displacement method. The hawthorn fruit samples were weighed and then placed in a pycnometer. The pycnometer was completely filled with toluene and weighed after drying the wall. This experiment was performed before and after drying the samples [12]. To reduce the effect of water penetration into the samples, the measurements were performed in the shortest possible time. The volume of the samples was then calculated using Equations (10) and (11) [26]:

(10) $$V=V_{f}-\frac{M}{\rho }$$

(11) $$M=m_{s}-(m_{spw}+m_{pw})$$

where $V_{f}$ is the pycnometer volume (cm^3^), M is the weight of added toluene to fill the pycnometer (g), $m_{s}$ is the weight of pycnometer plus the weights of the sample and toluene (g), $m_{spw}$ is the weight of pycnometer (g), $m_{pw}$ is the weight of the sample (g), and ρ shows the density of toluene (0.87 g/cm^3^ at 20 °C).

Shrinkage was calculated by substituting the sample volume into Equation (12) [27,28]:

(12) $$S_{b}=1-\frac{\beta_{f}}{\beta_{i}}\times 100$$

where $\beta_{i}$ is the initial volume (cm^3^); $\beta_{f}$ is the final volume (cm^3^).

#### 2.5.3. Rehydration Ratio (RR)

The samples were collected and cooled after reaching the desired moisture (0.2 on a dry basis). Then, they were kept in polyethylene bags in a dry and cool place to prevent moisture absorption until the rehydration ratio test. One week after the drying tests, the rehydration ratio tests were performed using a water bath. In this experiment, the dried samples were first weighed and then immersed in water at 50 °C for 30 min. After 30 min, the samples were removed from water, the surface water completely dried, and they were weighed with filter paper. The rehydration ratio was then calculated from Equation (13) [24]:

(13) $$RR=\frac{W_{r}}{W_{d}}$$

where *W_r_* is the mass of the dried sample (g); *W_d_* is the initial weight of the sample (g).

### 2.6. Bioactive Compounds Properties

#### 2.6.1. Measurement of the Antioxidant Capacity (AC) using the Diphenyl Picryl Hydrazine (DPPH) Method

To measure the content of phenolic compounds, flavonoids, and antioxidants, the extraction was first performed using the Lin et al. [29] method. The 10% methanolic extract was prepared by soaking and ultrasonic waves, and after the centrifugation (Sigma 1-14k, Germany), the extract was used in the next steps. For this purpose, 150 μL of the extracts were mixed with 2 mL of DPPH methanolic solution (0.01 M), and the absorption rate was then read after 30 min at room temperature in the dark using a spectrophotometer at 517 nm [30]. Total antioxidant activity was calculated as the percentage of DPPH radical scavenging with the following equation [31,32].

(14) $$I=\frac{\left(A_{i}-A_{t}\right)}{A_{t}}\times 100$$

where *I* is the antioxidant activity (%), and *A_i_* and *A_t_* are the absorbance values of the blank and sample, respectively.

#### 2.6.2. Total Phenolic Content (TPC)

The content of TPC was evaluated using the Folin–Ciocalteu colorimetric method. In this method, the content of TPCs was measured based on gallic acid, and the results were expressed as gallic acid equivalents. The color intensity generated at 760 nm was measured with a spectrophotometer. Using this method, 20 μL of the extract was added to the Folin–Ciocalteu reagent (15:1). After 5 min, 0.3 mL of 20% sodium carbonate solution was added to the solution, and the samples were kept in a bain-marie at 40 °C for 1 min after stirring. Then, the absorption of the samples was read with a spectrophotometer at 760 nm. The results were calculated in mg of gallic acid per 100 g of dry sample. Gallic acid was used as a standard to plot the calibration curve, and the results were calculated in mg of gallic acid per 100 g dw of the sample [33].

#### 2.6.3. Total Flavonoid Content (TFC)

TFC was measured by Beketove et al.’s [34] method. Initially, 4.5 mL of 90% ethanol, 200 μL of 2% aluminum chloride, and 100 μL of 33% acetic acid were added to 200 μL of the extracts, and after 30 min, the sample absorption was read at 414 nm using a spectrophotometer at a room temperature in the dark. Quercetin was used as a standard to plot the calibration curve, and the results were calculated in mg of quercetin per 100 g dw of the sample.

#### 2.6.4. Measurement of pH

A pH meter (Metrohm 827Ph Lab, Herisau, Switzerland) was used to measure pH. For this purpose, before drying, 5 g of the hawthorn fruit sample, ground by a home grinder, was dissolved in 100 mL of distilled water and stirred for 15 min by shaker at 50 rpm. Then, after resting the solution for 15 min, the pH was measured by the pH meter [35]. To measure the pH of the dried product, the samples were removed from the dryer after drying and pounded in a mortar. Then, the pH of the samples was measured and recorded at room temperature.

### 2.7. Statistics

The statistical analysis of the data was performed using a completely randomized design with the factor of drying methods in three replications. The comparison of means was made using the least significant difference (LSD) test at the 99% confidence level. SAS 9.4 software was used to perform statistical calculations

## 3. Results and Discussion

### 3.1. Drying Time

The effect of different drying methods on the time required for drying hawthorn fruits to achieve a moisture content of 0.2% on a dry basis was significant at the 1% level (Table 2). The times required for hawthorn fruits to be dried through hot-air, infrared–hot-air, microwave–hot-air, ultrasonic + hot-air, ultrasonic + microwave–hot-air, ultrasonic + infrared–hot-air, and freeze-drying methods were 450, 175, 70, 370, 45, 130, and 1280 min, respectively. As shown in Table 3, the shortest drying time was obtained by the combined ultrasonic + microwave–hot-air drying. Using ultrasonic pretreatment significantly reduced the drying time compared to drying without ultrasonic pretreatment. Mierzwa et al. [14] showed that using ultrasonic pretreatment to dry raspberries using hot-air, microwave, and microwave–hot-air drying reduces the drying time. By applying the cavitation process (asymmetric bursting of bubbles) near the food surface, ultrasonic pretreatment causes a rapid and eruptive flow of sound waves to the surface, forming microscopic channels in the samples by creating successive contractions and expansions. In addition, by increasing the duration of ultrasonic application, the channels expand, and the product has a spongy texture, facilitating the flow of water out of the product during the drying process through the created channels [11]. In addition, the microwave–hot-air method the water molecules inside the samples bipolar, followed by an increase in temperature inside the product and an increase in the internal vapor pressure in the sample. Eventually, the cellular texture of the sample swells, causing more pores in the samples [36]. A study on carrot drying was performed using different drying methods (i.e., microwave, hot air, infrared, microwave–hot-air, infrared–hot air) and ultrasonic pretreatment by Abbaspour-Gilandeh et al. [12]. The results showed that the ultrasonic pretreatment used for all drying methods reduced the drying time, which is consistent with the results of the present study. The longest drying time, 1280 min, was obtained via freeze-drying, while the shortest drying time of 45 min was obtained through microwave–hot-air drying with ultrasonic pretreatment. Qiu et al. [37] adopted the hot-air, freeze. and infrared–freeze-drying methods to dry the edible rose flower. The results showed that the freeze-drying method had the longest drying time.

A comparison between the different drying methods (i.e., hot air, vacuum, microwave, and freeze) was performed for carrot peels. The results of this study showed that the drying time was the highest in the freeze-drying method, while the drying time was the lowest for the microwave dryer. [38]. According to Table 3, for the infrared drying method, the drying time was significantly reduced compared to the hot-air-drying method. The times required using the infrared–hot-air and microwave–hot-air drying methods to reach a moisture content of 0.10 (d.b.) were 2.64 and 6.42 times faster than for hot-air drying, respectively (Table 3).

### 3.2. Specific Energy (SE)

Table 2 shows the analysis of variance related to the effect of different experimental methods on SE. Analysis of variance (Table 2) shows that there was a significant difference between different drying methods at the 1% level. Table 3 shows the effect of different experimental methods (i.e., hot air, infrared–hot air, microwave–hot-air, freeze, and ultrasonic pretreatment) on SE. According to the results, the lowest amount of SE was obtained by ultrasonic + microwave–hot-air drying (47.57 MJ/kg). The use of microwave–hot-air drying increased the heat gradient between the drying products, resulting in the increased rate of moisture evaporation [39]. Moreover, the results comparing the use or non-use of US showed that the use of ultrasonic reduced the amount of SE. Ultrasonic pretreatment causes more damage to the product texture, and the hard layer is not formed during the drying process in the pretreated product. Szadzińska et al. [40] showed that the use of ultrasonic pretreatment prior to drying raspberries in different drying methods (i.e., hot air and microwave) reduced SE compared to without pretreatment. In another study, Abbaspour-Gilandeh et al. [12] used ultrasonic pretreatment to dry carrots before drying (hot air, microwave, infrared, microwave–infrared, infrared–hot air). They found that using ultrasonic pretreatment reduced SE in all drying methods compared to non-use of ultrasonic pretreatment.

On the other hand, a higher SE of freeze-drying compared to other processes was attributed to the longer process time and lower temperature of the dryer chamber [41]. According to Liu et al. [41], who conducted a study on drying mushrooms via hot air, infrared, and freeze-drying showed that the freeze-drying had the highest amount of SE. Moreover, Shewale et al. [42], for drying apple; Nguyen and Le [38], for drying carrot peel; Saifullah et al. [43], for drying lemon myrtle showed that the highest amount of SE was obtained via the freeze-drying method compared to other drying methods.

### 3.3. Quality Properties

#### 3.3.1. Color

**Figure 1:**
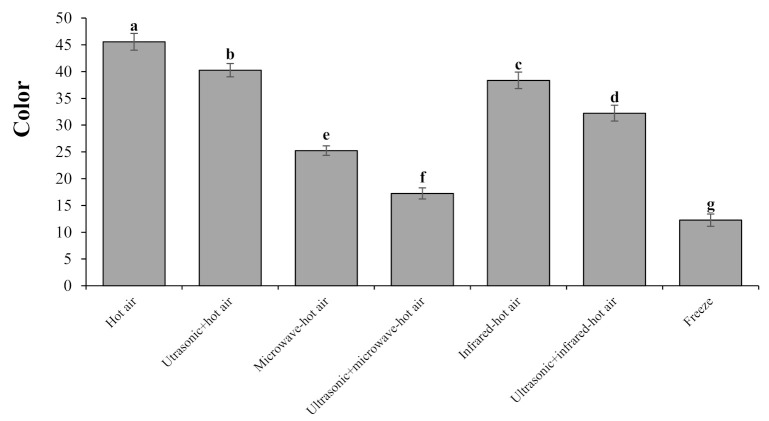
Color of dried hawthorns as affected by the drying method. Treatments included ultrasonic and hot air, microwave–hot air, infrared–hot air, and freeze-drying. Bars are means of three plants per replicates. Bars with different letters differ significantly from each other at *p* < 0.05 as determined by LSD test.

The examination of the analysis of variance (Table 2) for the factor of color that there is a significant difference between different drying methods at the 1% level (*p* < 0.01). Comparing the results (Figure 1) obtained from color changes among the different drying methods showed that the highest and lowest changes in this parameter occurred in the hot-air and freeze-drying methods, respectively. The reason for the high color changes in the hot-air drying can be the decomposition of pigments and the enzymatic and non-enzymatic browning reactions due to the prolonged exposure to heat, which destroys the physical quality of the sample and increases the color changes of hawthorn fruits. In addition, the reason for the low rate of color change in the freeze-dried samples was that the surface contact of the product with oxygen was reduced due to the vacuum condition during the drying, and the product did not have the prolonged exposure to the heat and oxidation of pigments; [4]. Feng et al. [44] used the freeze, hot-air, infrared, relative humidity drying (RHD), and pulsed vacuum drying (PVD) methods to study garlic color changes during the drying process and concluded that the least amount of color change was obtained during the freeze-drying method. They stated that the fewer color changes in the freeze method were due to the low heat and vacuum space. On the other hand, among the heat-dried hawthorn fruit samples, the microwave–hot-air-drying method with ultrasonic pretreatment had the least amount of color changes (i.e., the original color was much more preserved). This could be significantly (*p* < 0.01) related to the shortest drying time [45]. Reducing the drying time and increasing the heat reduces the enzymatic activity, and color changes during the drying process are made by ultrasonic + microwave–hot-air drying [44].

Abbaspour-Gilandeh et al. [12] examined carrots drying with the use of different methods (i.e., hot air, microwave, infrared, microwave–infrared, and infrared–hot air) by ultrasonic pretreatment. The microwave–hot-air-drying method with ultrasonic pretreatment resulted in the highest drying quality in terms of color compared to other drying methods. In addition, Szadzińska et al. [46] reported that dried beetroot resulting from microwave–hot-air drying with ultrasonic pretreatment had fewer color changes than drying with the microwave–hot-air, hot-air, and hot-air with ultrasonic pretreatment methods. The infrared–hot-air drying further darkened the color of hawthorn fruit compared to the infrared–hot-air drying with ultrasonic pretreatment, where the dark color after the drying process was due to the fact of chlorophyll degradation [47].

#### 3.3.2. Shrinkage

**Figure 2:**
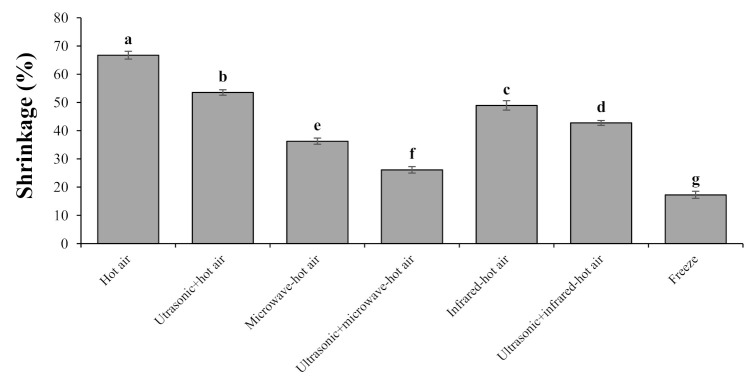
Shrinkage of dried hawthorns affected by different drying methods. Bars with different letters differ significantly from each other at *p* < 0.05 as determined by LSD test.

The variations in the shrinkage showed that the highest shrinkage rate was related to hot-air drying (66.75%), and the lowest rate was related to the use of the freeze-drying method (17.21%) (Figure 2). One of the most important reasons for the obtained results was that using the prolonged heating method caused a large amount of moisture to escape during of the drying process, which induces thermal stress in the samples, followed by the increased shrinkage in the samples [27]. The examination of shrinkage changes in the freeze-drying method showed that the shrinkage rate for this method was approximately from 8.89% to 49.54% lower than the other six methods used. One of the main reasons for the low shrinkage rate in the freeze-drying method was that due to the low drying temperature, where the moisture content of the samples was converted into solid crystals, followed by the sublimation of solid crystals with a decrease in pressure during the drying process. Therefore, after the sublimation of water during the drying process, the product almost retained its original structure and became porous, and the solid network (dried samples) could retain this porous structure to some extent [8,48]. Similar results have been reported by Qiu et al. [8] for drying rose flower chips using different methods (i.e., freeze, pulse spouted-vacuum–microwave freeze, vacuum–microwave freeze, and microwave-assisted vacuum drying). They showed that the lowest rate of shrinkage was obtained using the freeze-drying method at a significant level (*p* < 0.05). In addition, in another study on drying broccoli using different methods (i.e., freeze, hot air, microwave–vacuum, vacuum, microwave–vacuum–hot air, microwave–vacuum–vacuum), the lowest and highest shrinkage changes were obtained through the freeze and hot-air-drying methods at a significant level (*p* < 0.05), respectively [4]. The results of examining different drying methods (i.e., hot air, freeze, microwave–vacuum, vacuum, microwave–vacuum–hot air, microwave–vacuum–vacuum) on cabbage showed that the highest rate of shrinkage was obtained using the hot-air-drying method, and the lowest rate of shrinkage was obtained by the freeze-drying method [27]. Similar results were reported by Jiang et al. [16], who dried okra snacks. The results of the various previous studies are consistent with the results of the present study.

The results obtained from using the microwave–hot-air method compared to the other two drying methods (hot air and infrared–hot air) showed that this method reduced the shrinkage rate. This is because the use of microwave radiation causes cell expansion (swelling effect) and, thus, reduces shrinkage due to the pressure of water vapor inside the hawthorn fruit [4]. Similar results have been reported by Abbaspour-Gilandeh et al. [47] for drying *Pistacia atlantica*, Dehghannya et al. [49] for drying potato, and Aydogdu et al. [50] for drying eggplant. However, when using ultrasonic pretreatment, the amount of change in shrinkage was reduced in all drying methods. The reason for this phenomenon is that when using ultrasonic pretreatment, mass transfer becomes faster and, thus, moisture leaves the product faster, causing collapse and stress in the cell structure, and the void of moisture causes the shrinkage of hawthorn cells, which reduces the volume and causes shrinkage [12]. Similar results have been reported by Szadzińska et al. [40] for raspberries and Abbaspour-Gilandeh et al. [12] for carrots, which show that the use of ultrasonic pretreatment reduces shrinkage.

#### 3.3.3. Rehydration Ratio (RR)

**Figure 3:**
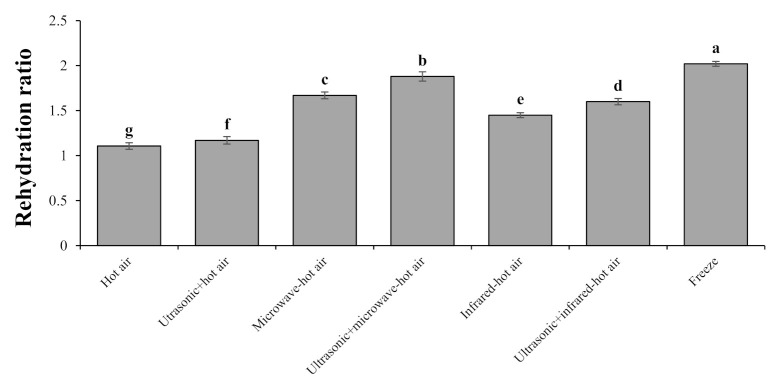
Rehydration ratio of dried hawthorns affected by different drying methods. Bars with different letters differ significantly from each other at *p* < 0.05 as determined by LSD test.

One of the most obvious changes in dry foods is a decrease in the RR power. It can be stated that the main factor in reducing the power of RR is the shrinkage and loss of cells and capillary tubes inside the food texture [24]. The examination of the analysis of variance (Table 2) for the RR indicated that there was a significant different effect among drying methods at the 1% level. According to Figure 3, the highest RR was 2.02 for freeze-drying. The food water sublimation resulted in a porous and unshrunk structure, and the dried hawthorn fruit had a high rate of rehydration during freeze-drying due to the low shrinkage rate [42]. Zhang et al. [51] examined the drying of golden pompano using the freeze, microwave, hot-air, and microwave–vacuum methods. They showed that the highest amount of RR was obtained in freeze-drying. Moreover, the lowest RR in hot-air drying was 1.09. In the case of hot-air drying, the lowest RR occurred due to the long drying time, large changes in food structure, much higher shrinkage, and much lower porosity [52].

To calculate the RR of peach leather drying, Azam et al. [6] compared different drying methods including the hot-air, infrared, microwave–hot-air, and hot-air-assisted radio-frequency drying methods. They showed that hot-air drying was less capable of determining the RR than other methods. Shewale et al. [42] showed that the highest and lowest RR for drying apples using different drying methods (i.e., radio frequency, low-humidity air drying, hot-air drying, and freeze-drying) were significantly (*p* < 0.05) obtained for freeze and hot-air drying methods. They stated that the reason for higher RR in freeze-drying is the porous structure created during freeze-drying. This review of studies shows that the results of this study are similar to previous research conducted on other products. The ultrasonic-treated samples showed a higher RR compared to the samples without ultrasonic treatment. This was due to the formation of microscopic channels created by ultrasonic waves, which helps the mass transfer during the drying process by various methods. Therefore, during the rehydration process, the samples absorbed more water in a shorter amount of time. Similar effects of ultrasonic pretreatment on RR have been reported for carrots [12,53], apples [54], and okra snacks [16], which increases the RR properties compared to the samples without ultrasonic pretreatment.

### 3.4. Bioactive Compounds

#### 3.4.1. Antioxidant Capacity (AC)

The results from the comparison of mean values indicated the significant effect of the drying method on the AC of hawthorn fruit. The mean values of antioxidants for different drying methods ranged from 5.98% to 30.69% (33.32% for fresh samples). The highest percentage of activity was obtained by the freeze-drying method (*p* < 0.01, Table 4). Freeze-drying had the highest AC, which was due to the lack of thermal degradation of heat-sensitive compounds. Liu et al. [3] conducted drying of hawthorn fruit in different drying methods (i.e., hot air, microwave, solar, and freeze) and showed that the highest amount of AC was obtained using freeze-drying. Turkmen et al. [55], Izli et al. [7], Kaveh et al. [56], and Szychowski et al. [5] investigated the AC when drying cherry laurel, kiwifruit, green pea, and quince using different drying methods, respectively, and found that the AC in the freeze-dried samples was higher than other drying methods, which was consistent with the results of this study. The results show that the lowest amount of AC was obtained for hot-air drying. In the hot-air-drying method, as the samples dry at a slow rate, the long drying time causes the loss of bioactive compounds and, as a result, the amount of AC in this method was the lowest one. The bioactive compounds are known to be responsible for the AC of foods [57]. Szychowski et al. [5] showed that the lowest amount of AC for drying quince fruit among different drying methods (i.e., hot air, freeze, and vacuum–microwave) was obtained for the hot-air drying. They stated that the low amount of AC was due to the intensive oxidation during the relatively long exposure to hot air. Many researchers have shown that the use of the hot-air-drying method reduces AC [4,36,58,59].

Table 5 shows that for the heating methods, the microwave–hot air method with ultrasonic pretreatment had the highest amount of AC, because microwave energy, drying temperature, and ultrasonic power may damage the cell structure and then release the compounds that may be electron–donor hydrogen atoms and, thus, inhibit free radicals [60]. The use of ultrasonic pretreatment before drying also increases AC, because ultrasonic inhibits higher free radicals [13]. The positive effects of ultrasonic on raising antioxidants have been reported in plants such as papaya [61], okra [13], and potato [62].

#### 3.4.2. Total Phenol Content (TPC)

As can be seen in Table 5, the highest number of phenolic compounds was obtained by using the freeze-drying method (*p* < 0.01, Table 4). This was probably due to the lower degradation of heat-sensitive phenolic compounds as a result of the low ambient temperature during the freeze-drying process [13]. The study on different methods of drying persimmons showed that the highest TPC was obtained after drying by the freeze-drying [59]. The study of drying broccoli using different methods showed that freeze-drying can maximize the amount of TPC [4].

The order of the effect of different drying methods on the total phenolic content is as follows: fresh > freeze ˃ ultrasonic + microwave–hot air > microwave–hot air > ultrasonic + infrared–hot air > hot air–infrared > ultrasonic + hot air ˃ hot air. According to the results, the lowest amount of TPC was obtained for the hot-air drying (29.56 mg GAE/gdw). In this method, the cell structure is mostly damaged, which is due to the long exposure of the product to the heat. In other studies, such as on *Pistacia atlantica* [47], cabbage [27], and hawthorn fruit [3], the hot-air method had the lowest amount of phenolic content compared to other drying methods, which is similar to the results of this study.

Among the other six drying methods, the microwave–hot-air drying method with ultrasonic pretreatment showed the least degradation of phenolic compounds due to the fact of less damage to the cell structure caused by the ultrasonic pretreatment. According to Table 5, it can be seen that ultrasonic pretreatment along with different drying methods improves the TPC. Therefore, the microwave + hot-air drying with ultrasonic pretreatment had the highest amount of TPC compared to other thermal drying methods. Xu et al. [13] reported that ultrasonic pretreatment improves the TPC of okra fruit. Rashid et al. [62] also reported that the use of ultrasonic pretreatment to obtain TPC in potatoes leads to cell breakage, thus releasing phenolic compounds from the membrane matrix, which usually occurs during the decomposition of cell components.

Lim and Murtijaya [63] reported that the heat generated by microwave radiation is very rapid and intense, which can cause serious damage to polyphenolics. In addition, polyphenol oxidase and peroxidase activities during the drying process can cause loss of TPC, while Xu et al. [4] observed that broccoli polyphenolics improved during the microwave process. They explained that there is a special energy during microwave–hot-air drying process that can lead to the decomposition of cellular elements and cause more polyphenolics to be secreted from the product texture. However, TPC varies irregularly in different plant species in different drying methods [64].

#### 3.4.3. Total Flavonoid Content (TFC)

According to the results of the analysis of variance in Table 4, the effect of different drying methods on the content of TFC compounds in hawthorn fruits was significant at the 1% level. The effect of the different methods of thermal drying on the extraction rate of TFC compounds over time with and without ultrasonic pretreatment is shown in Table 5. The highest and lowest amounts of TFC were obtained in the freeze (65.93 mg QE/gdw) and hot-air (27.26 mg QE/gdw) drying methods, respectively. The results of drying horseradish leaves pomace [60] and apple [42] were similar to these results. Shewale et al. [42] stated that the low number of flavonoids in hot-air drying can be attributed to the oxidative degradation of phenolic.

The TFCs of hawthorn fruit extract in the microwave–hot-air, ultrasonic + microwave–hot-air, infrared–hot-air, ultrasonic + infrared–hot-air, and ultrasonic+ hot-air-drying methods were 46.39, 51.17, 36.49, 39.43, and 28.56 mg-QE/g d.w., respectively. Therefore, the order of effect of the different drying methods on the TFC is as follows: fresh > freeze ˃ ultrasonic + microwave–hot air > microwave–hot air > ultrasonic + infrared–hot air > infrared–hot air > ultrasonic + hot air ˃ hot air. From the obtained results, it can be seen that the amount of flavonoid content obtained from drying hawthorn fruit is higher [2].

From Table 5, it can be seen that the use of ultrasonic pretreatment before drying had a significant effect on the increased amount of hawthorn fruit’s TFC, because ultrasonic causes the release, alteration of bound phenolics, and inactivation of some enzymes that act on polyphenolics [65]. The TFC values in the present study can be compared with the research on flavonoid extraction by ultrasonic in sweet potatoes [62], cranberry snacks [11], okra [13], and hawthorn [2]. They also concluded that the use of ultrasonic pretreatment before drying increased the TFC, which is consistent with the results of this study.

Moreover, due to the high temperature, microwave–hot-air drying may be able to decompose covalent bonds and, thus, release biological compounds, such as flavonoids, from the polymer replication, resulting in a higher amount of TFC [66].

#### 3.4.4. pH

**Figure 4:**
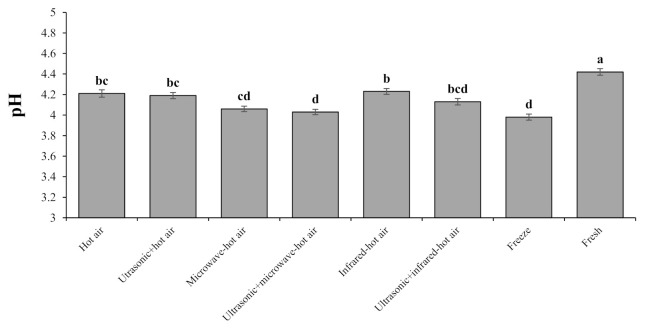
pH of dried quinces affected by different drying method. Bars with different letters differ significantly from each other at *p* < 0.05 as determined by LSD test.

The examination of the analysis of variance (Figure 4) for pH indicates that there was a significant difference among the different drying methods at the 1% level. The results of the average pH of hawthorn fruit extract are shown in Figure 4. As can be seen, the highest pH was obtained by the combined infrared–hot-air (4.23) and hot-air (4.21) drying methods. According to the diagram, increasing the drying time increased the pH, which can be attributed to the type of compounds extracted from hawthorn fruit and also the further degradation of cell walls over time. The increase in pH could be due to the Maillard reactions and the high concentration of soluble solids during the prolonged drying [67]. The lowest pH was achieved by freeze-drying (3.98) and combined microwave–hot-air drying with ultrasonic pretreatment (4.03). The chemical compounds resulted from the browning reaction included soluble and insoluble polymers that were formed in the places where a reducing sugar combines with a protein amino acid or other nitrogenous compounds. In this reaction, due to the loss of amino groups and the formation of organic acids, the food pH decreases [68].

## 4. Conclusions

The results of drying hawthorn fruit using different drying methods (i.e., hot air, microwave–hot air, infrared–hot air, freeze, ultrasonic + hot air, ultrasonic + microwave–hot-air, ultrasonic + infrared–hot air) showed that the drying time varied from 50 to 1280 min. The hot-air drying, hot-air drying with ultrasonic pretreatment, and ultrasonic–hot-air drying methods resulted in longer drying times and higher specific energies, color changes, and shrinkage. However, the microwave–hot-air, ultrasonic + microwave–hot-air, and freeze-drying methods made the hawthorn fruit smooth and shrunken in appearance with much more nutritional value. Furthermore, the hot-air, and infrared–hot-air drying methods led to high losses of bioactive compounds, while the ultrasonic + microwave–hot-air and freeze-drying methods released more biological compounds. The microwave + hot-air drying with ultrasonic pretreatment seems to be a suitable method for drying hawthorn fruits due to the appropriate drying time, lower specific energy, color change, and shrinkage and higher rehydration ratio, antioxidant capacity, total phenol content, and total flavonoid content. The results of this study provide useful data for producers, suppliers, and consumers of hawthorn fruit.

## Figures and Tables

**Table 1:** Specific energy consumption equations for different dryers and ultrasonic pretreatment.

Energy Consumption at Dryer	Reference
E_t_ (hot air) = Equations (2) + Equations (3)	[21]
E_t_ (microwave–hot-air) = Equations (2) + Equations (3) + Equations (5)	[22]
E_t_ (infrared–hot air) = Equations (3) + Equations (4)	[21]
E_t_ (ultrasonic + hot air) = Equations (3) + Equations (4) + Equations (7)	[23]
E_t_ (ultrasonic + microwave–hot-air) = Equations (2) + Equations (3) + Equations (5) + Equations (7)	[12]
E_t_ (ultrasonic + infrared–hot air) = Equations (3) + Equations (4) + Equations (7)	[12]
E_t_ (freeze) = Equations (8)	[4]

**Table 2:** Analysis of variance (ANOVA) for the drying methods on drying time, SE, color, shrinkage, and RR.

SOV	df	Time	SE	Color	Shrinkage	RR
Drying method	6	562,525.00 **	44,198.96 **	461.47 **	844.35 **	0.348 **
Error	14	136.87	32.71	0.45	1.94	0.00
CV		3.24	3.47	0.70	3.34	1.75

** Significant at the 1% probability level.

**Table 3:** Influence of different drying methods on drying time and specific energy of hawthorn.

Drying Method	Time (min)	SE (MJ/kg)
Hot air	450 ± 7.58 b	245.70 ± 7.58 b
Microwave–hot-air	70 ± 1.5 f	64.95 ± 5.12 f
Infrared–hot air	175 ± 3.0 d	122.16 ± 6.77 d
Ultrasonic + hot air	370 ± 6.0 c	194.80 ± 6.98 c
Ultrasonic + Microwave–hot-air	45 ± 1.0 g	47.57 ± 4.44 g
Ultrasonic + Infrared–hot air	130 ± 3.0 e	89.95 ± 5.99 e
Freeze	1280 ± 11.5 a	388.03 ± 9.10 a

Values expressed as mean ± standard error (*n* = 3), different letters in each column indicate a significant difference at *p* ≤ 0.05 levels.

**Table 4:** Analysis of variance (ANOVA) for drying methods on AC, TPC, TFC, and pH.

SOV	df	AC	TPC	TFC	pH
Drying method	7	825.95 **	315.25 **	1009.75 **	0.058 **
Error	16	0.40	0.62	1.69	0.000
CV		1.37	4.07	2.38	2.12

** Significant at the 1% probability level.

**Table 5:** Influence of different drying methods on AC, TPC, and TFC of hawthorn.

Drying	AC (%)	TPC (mg GAE/gdw)	TFC (mg QE/gdw)
Fresh (no drying)	33.32 ± 1.20 a	79.82 ± 2.42 a	72.72 ± 2.88 a
Hot air	5.98 ± 0.94 h	29.56 ± 1.99 h	27.26 ± 2.16 h
Microwave–hot air	22.23 ± 0.85 d	58.51 ± 2.32 d	46.39 ± 3.25 d
Infrared–hot air	11.83 ± 1.10 f	40.51 ± 3.21 f	36.49 ± 2.09 f
Ultrasonic + hot air	9.45 ± 1.15 g	36.28 ± 2.09 g	28.56 ± 3.52 g
Ultrasonic + microwave–hot air	26.38 ± 1.31 c	68.22 ± 2.28 c	51.17 ± 2.48 c
Ultrasonic + infrared–hot air	14.79 ± 0.82 e	52.56 ± 3.19 e	39.43 ± 1.99 e
Freeze	30.69 ± 1.13 b	73.07 ± 2.95 b	65.93 ± 2.02 b

Values expressed as mean ± standard error (*n* = 3), different letters in each column indicate significant difference at the *p* ≤ 0.05 levels. GAE = gallic acid, QE = quercetin.

## Data Availability

The data presented in this study are available upon request from the corresponding author.
